# Low prevalence of archived integrase strand transfer inhibitors resistance associated mutations in Botswana before the roll out of dolutegravir based first line antiretroviral therapy

**DOI:** 10.3389/fmicb.2024.1482348

**Published:** 2024-10-24

**Authors:** Dorcas Maruapula, Doreen Ditshwanelo, Marea N. Pema, Ontlametse T. Bareng, Wonderful T. Choga, Natasha O. Moraka, Patrick T. Mokgethi, Kaelo K. Seatla, Catherine K. Koofhethile, Boitumelo J. Zuze, Tendani Gaolathe, Molly Pretorius-Holme, Kebaneilwe Lebani, Joseph Makhema, Vlad Novitsky, Roger Shapiro, Shahin Lockman, Sikhulile Moyo, Simani Gaseitsiwe

**Affiliations:** ^1^Botswana Harvard Health Partnership, Gaborone, Botswana; ^2^Faculty of Health Sciences, Medical Laboratory Sciences, University of Botswana, Gaborone, Botswana; ^3^Department of Biological Sciences, University of Botswana, Gaborone, Botswana; ^4^Department of Immunology and Infectious Diseases, Harvard University T.H Chan School of Public Health, Boston, MA, United States; ^5^Department of Biological Sciences and Biotechnology, Botswana International University of Science and Technology, Palapye, Botswana; ^6^Division of Infectious Disease, Brigham and Women’s Hospital, Boston, MA, United States; ^7^School of Health Systems and Public Health, Faculty of Sciences, University of Pretoria, Pretoria, South Africa

**Keywords:** Botswana, HIV, integrase strand transfer inhibitor, HIV drug resistance mutations, antiretroviral therapy

## Abstract

**Background:**

We evaluated the prevalence of archived proviral drug resistance mutations (DRMs) associated with resistance to integrase strand transfer inhibitors (INSTIs) shortly before Botswana transitioned in 2016 to using dolutegravir (DTG)-based antiretroviral treatment in first-line regimens.

**Methods:**

We used the Stanford University HIV drug resistance database to analyze INSTI-resistance associated mutations (RAMs) in a large representative population-based cohort of adults recruited in 30 geographically dispersed communities as part of the Botswana Combination Prevention Project (BCPP) cohort from 2013 to 2018. A total of 5,144 HIV-1 proviral DNA sequences were included in our analysis; 1,281 sequences were from antiretroviral therapy (ART)-naïve individuals and 3,863 sequences were from non-nucleoside reverse transcriptase inhibitor (NNRTI) ART-experienced individuals. None of the sequences were from DTG-ART experienced participants.

**Results:**

The overall prevalence of major INSTIs DRMs was 1.11% (95% CI 0.82–1.39%). The prevalence of INSTI DRMs in ART-naïve individuals was 1.64% (21/1,281) and 0.93% (36/3,863) in ART-experienced individuals. Major INSTI-RAMs detected in ART-naïve individuals were E138K (2/1,281; 0.16%), G140R (8/1,281;0.62%), E92G (2/1,281;0.16%), R263K (5/1,281; 0.4%), N155H (1/1,281; 0.08%), P145S (1/1,281;0.008%). Among the ART-experienced individuals, major INSTI RAMs detected were E138K (4/3,863; 0.10%), G140R (25/3,863;0.65%), G118R (2/3,863, 0.05%), R263K (4/3,863, 0.10%), T66I (1/3,863;0.03%), E138K + G140R (1/3,863, 0.03%|), G140R + R263K (1/3,863, 0.03%). High-level resistance to cabotegravir (CAB), elvitegravir (EVG), and raltegravir (RAL) was detected in 0.70, 0.16 and 0.06% of the individuals, respectively. Notably, bictegravir (BIC) and dolutegravir (DTG) showed no high-level resistance.

**Conclusion:**

The overall prevalence of archived INSTI RAMs in Botswana was low prior to transitioning to first-line DTG-based ART regimens, and did not differ between ART-naïve and ART-experienced individuals. Ongoing surveillance of INSTI DRMs in Botswana will allow for re-assessment of INSTI resistance risk following nationwide DTG rollout.

## 1 Introduction

Botswana, with an HIV prevalence of 20.8% in adults and 0.8% in children (Mine et al., 2024), was one of the first countries in sub-Saharan Africa to address this high burden by rolling out universal antiretroviral therapy (ART) in 2002 and dolutegravir (DTG)-based ART in June 2016. In Botswana, the HIV epidemic is predominantly caused by single viral subtype, which is the HIV-1 Subtype C (HIV-1C). HIV-1C is mainly prevalent in Southern Africa and has been the main subtype in Botswana for several years (Novitsky et al., 2020; Moyo et al., 2019). Botswana’s early rollout of DTG-based ART makes it an ideal country to perform surveillance for the development of integrase strand transfer inhibitor (INSTI) resistance mutations. As with all other antiretroviral (ARV) classes, INSTI-resistance associated mutations (RAMs) have been identified and are a major challenge for the success of HIV treatment using an INSTI-containing regimen (Seatla et al., 2021; Steegen et al., 2019). With the widespread use of INSTI-based ART, it is important to have good surveillance of INSTI-RAMs so that appropriate action can be taken timely if there is an upsurge of INSTI RAMs.

To date, there has not been any large study exploring the prevalence of INSTI-RAMs in people living with HIV (PLWH) in Botswana before the roll out of INSTI-based first-line ART. Analyzing INSTI drug resistance mutations (DRMs) in INSTI-naïve individuals provides valuable insights into historical trends and the evolution of INSTI resistance, essential for anticipating future challenges. Analyzing past DRMs helps predict future resistance patterns, crucial for public health planning, maintaining the effectiveness of antiretroviral therapies, and guiding resource allocation and policy decisions. Additionally, understanding INSTI-RAMs is vital because they can significantly impact the efficacy of other integrase inhibitors, including cabotegravir (CAB), which is used in both pre-exposure prophylaxis (PrEP) and as part of long-acting injectable (LAI) regimens.

We sought to determine the prevalence of INSTI-RAMs in a large cohort of PLWH in Botswana before the roll out of DTG-based first-line ART in Botswana. The data generated here forms a reference point on the baseline prevalence of INSTI-RAMs in Botswana.

## 2 Materials and methods

### 2.1 Study population

This was a cross-sectional study utilizing provirus sequences from a previously conducted study known as the Botswana Combination Prevention Project (BCPP; Makhema et al., 2019). BCPP was a community randomized trial conducted from 2013 to 2018, which examined the impact of community-based prevention interventions on HIV incidence in 30 communities in northern, central and southern parts of Botswana. The study involved 15 intervention communities and 15 control communities (Supplementary Table 1), focusing on people living with HIV aged 16–64 years (Makhema et al., 2019). These communities are well described elsewhere (Makhema et al., 2019). Most participants in the BCPP cohort were on antiretroviral therapy and had achieved undetectable viral load (Gaolathe et al., 2016).

### 2.2 Selection of study participants

**Figure 1:**
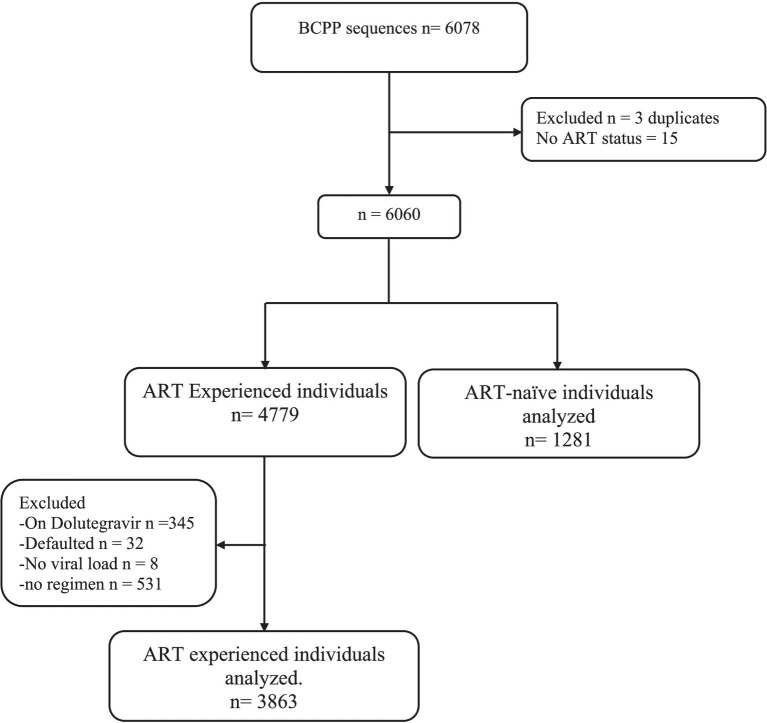
Study schema showing participants selection for the analysis.

A total of 5,144 out of 6,075 available HIV-1 near full-length proviral DNA sequences generated using next-generated sequencing from the BCPP cohort (Moyo et al., 2019) were utilized in this analysis (Figure 1). Proviral sequences for participants on second-line DTG-containing regimen were excluded from our analysis. Among the proviral sequences included, 1,281 were from ART-naïve individuals and 3,863 were from ART-experienced individuals (primarily efavirenz/lamivudine/tenofovir). We defined viral suppression as individuals with viral load ≤400copies/ml, as per Botswana’s 2016 HIV treatment guidelines definition (MoH, 2016).

### 2.3 Apolipoprotein B mRNA editing enzyme, catalytic polypeptide-like (APOBEC)-induced hypermutations

The sequences were aligned with Multiple Alignment using Fast Fourier (MAFFT) within the HIV align tool in the Los Alamos database.^1^ The aligned sequences for both ART-naïve and ART-experienced individuals were screened for hypermutations using the online hypermut 2.0 program (available at https://www.hiv.Ianl.gov/content/sequence/HYPERMUT/hypermut.html; Rose and Korber, 2000). Mutations potentially arising from hypermutations were identified and subsequently excluded from the analysis.

### 2.4 Integrase strand transfer inhibitor resistance analysis

INSTI-RAMs were identified using the Stanford HIV Drug Resistance Database (HIVdb v9.0; https://hivdb.stanford.edu/hivdb/by-mutations). INSTI-RAMs were categorized as major resistance mutations and accessory resistance mutations according to the Stanford HIV Drug Resistance Database. The assessment of INSTI resistance levels was conducted utilizing the Stanford HIV Drug Resistance database according to resistance penalty score. The resistance levels were stratified into different categories, including low-level resistance, potential low-level resistance, intermediate resistance, and high-level resistance.

### 2.5 Statistical analysis

Statistical analyses were conducted using Stata SE 15 (Stata Corp, College Station, TX, United States). Baseline characteristics of the study participants were summarized using descriptive statistics. Statistical differences between ART-naïve and ART-experienced groups were assessed using Fisher’s exact test, the Chi-squared test, and the Mann–Whitney U-test. Categorical variables (gender) were reported as percentages and continuous variables (VL and age) as medians with interquartile ranges. The statistical significance was calculated using the Fisher exact test or Chi-square for categorical variables and the Mann–Whitney test for continuous variables. The comparison of INSTI RAMs was compared among ART-naïve group and ART-experienced group using Chi-square test. *p*-values less than 0.05 were considered statistically significant.

## 3 Results

### 3.1 Characteristics of study population

Of the 5,144 individuals included in our study, majority (70.2%) of the study participants were female (3,610/5,144) and the median (interquartile, IQR) ages for females and males were 39 (32–47.3) and 42 (35–49.8) years, respectively (Table 1). The median plasma viral load was 4.3 and 1.6 log10 RNA copies/mL for ART naïve and experienced, respectively. We included individuals naïve to integrase transfer strand inhibitors (INSTIs), stratified into 1,281 who had never been exposed to any ART (ART-naïve) and 3,863 who had been exposed to nucleoside reverse transcriptase inhibitors (NRTI), non-nucleoside reverse transcriptase inhibitors (NNRTI) based regimen or protease inhibitor (PI) based regimens (ART-experienced).

**Table 1:** Demographic characteristics of study population.

Parameter	Total (*N* = 5,144)	ART-naïve individuals (*n* = 1,281)	ART-experienced individuals (*n* = 3,863)	
			ART-suppressed (*n* = 3,735)	ART-unsuppressed (*n* = 128)
Sex (*n*, %)				
Female	3,610 (70.2)	856 (66.8)	2,674 (71.6)	80 (62.5)
Male	1,534 (29.8)	425 (33.2)	1,061 (28.4)	48 (37.5)
Age, years, median (Q1, Q3)				
Female	39 (32, 47.3)	33 (26.6, 42)	40.7 (34, 48.2)	38.8 (28, 45)
Male	42 (35, 49.8)	36 (29.9, 42.2)	44 (38, 51.7)	32.5 (26, 39)
ART regimens used (*n* = 3,863)				
EFV	2,544 (65.9%)	NA	2,462 (65.9%)	82 (64.1%)
NVP	1,098 (28.4%)		1,073 (28.7%)	25 (19.5%)
LPV	212 (5.5%)		192 (5.1%)	20 (15.6%)
RTV	9 (0.2%)		8 (0.2%)	1 (0.8%)

ART, antiretroviral therapy; N, population size; EFV, efavirenz-based therapy; NVP, nevirapine; LPV, lopinavir; RTV, ritonavir.

### 3.2 Overall prevalence of INSTI RAMs

**Figure 2:**
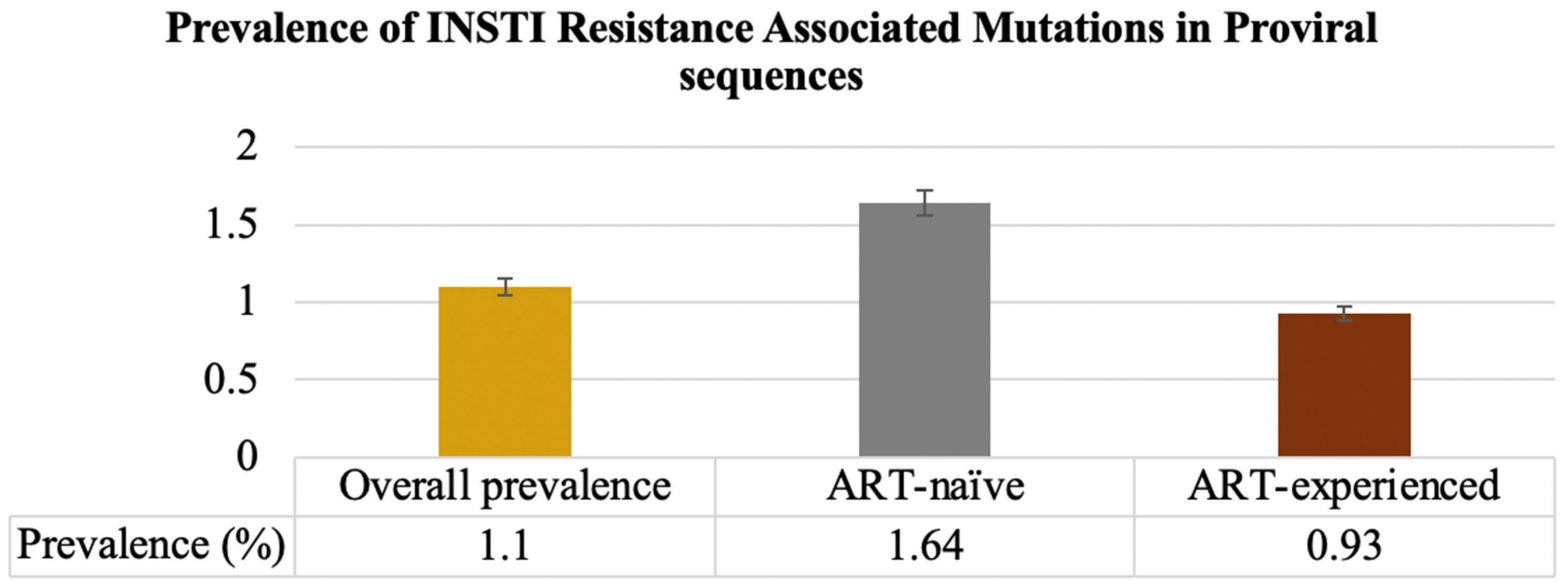
Prevalence of INSTI RAMs in INSTI-naïve (ART-naïve and ART-experienced individuals). The overall prevalence of INSTI RAMs was 1.1%. Among ART-naïve individuals, the prevalence of at least one major INSTI RAMs was 1.64%. For individuals on ART, the prevalence of INSTI RAMs was 0.93%.

Prior to excluding hypermutants, 2.9% (37/1,281) of ART-naïve individuals had INSTI-RAMs, while 4.2% (164/3,863) of ART-experienced individuals had INSTI-RAMs. After excluding hypermutants, the frequency of at least one major INSTI resistance mutation was 1.64% (21/1,281; 95% CI: 0.94–2.3) among ART-naïve individuals and 0.93% (36/3,863; 95% CI: 0.63–1.2) among ART-experienced individuals (*p* = 0.03; Figure 2). Among the ART-experienced group, those with virological suppression had INSTI RAMs, while none of the ART-experienced individuals with virological failure had INSTI RAMs (Supplementary Figure 1). There was no significant difference in the prevalence of accessory mutations observed among ART-naïve group (0.16%, 2/1,281) and ART-experienced group (0.16%, 6/3,863; *p* = 0.06). The following accessory mutations were observed: A128T and G163R in ART-naïve individuals (one instance each), and G163R in six ART-experienced individuals.

### 3.3 INSTI resistance among ART naïve individuals

**Figure 3:**
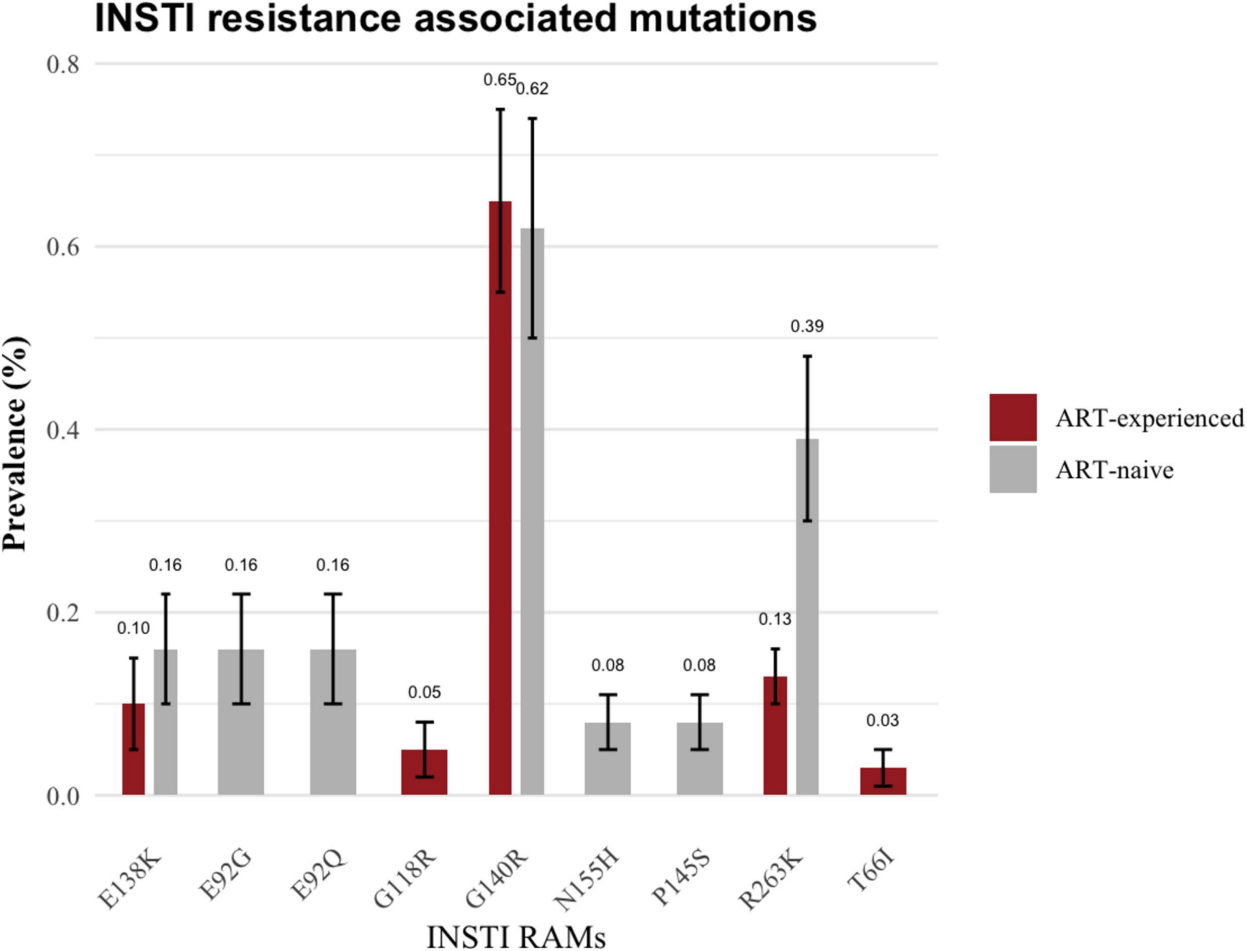
Frequency of integrase strand transfer inhibitor resistance associated mutations.

The specific resistance mutations identified included E138K (*n* = 2; 0.16%), G140R (*n* = 8; 0.62%), E92G (*n* = 2; 0.16%), E92Q (*n* = 2; 0.16%), R263K 0.4% (*n* = 5; 0.39%), N155H (*n* = 1; 0.08%) and P145S (*n* = 1; 0.08%). The INSTI-RAMs in these ART-naïve group are shown in Figure 3. Moreover, two ART-naïve participants exhibited INSTI accessory mutations, A128T and G163R.

### 3.4 INSTI resistance among ART-experienced individuals

Among ART-experienced / INSTI-naïve participants, 0.93% (36/3,863) harbored major mutations associated with INSTI. The prevalence of these major mutations was as follows: G140R (0.65%, 25/3,863), E138K (0.10%, 4/3,863), G118R (0.05%, 2/3,863), R263K (0.13%, 5/3,863) and T66I (0.03%, 1/3,863; as illustrated in Figure 3). Additionally, G163R INSTI accessory mutation was present in 0.16% (6/3,863) of the ART-experienced sequences.

### 3.5 Resistance levels to INSTIs

Among all analyzed sequences, high-resistance to CAB, elvitegravir (EVG) and raltegravir (RAL) was observed in 0.70% (36/5,144), 0.16% (8/5,144), and 0.06% (3/5,144), respectively (Table 2; Supplementary Figure 2). Notably, there was no high-resistance identified for bictegravir (BIC) and DTG (Table 2).

**Table 2:** INSTI major drug resistance detected and resistance levels.

DRMs	INSTI DRMs in ART naives individuals (*n* = 1,281)	INSTI DRMs in ART experienced individuals (*n* = 3,863)	BIC	CAB	DTG	EVG	RAL
E138K	2	5	LR	LR	LR	LR	LR
G140R	8	27	LR	HR	LR	IR	IR
E92G	2	0	S	LR	S	IR	LR
E92Q	2	0	LR	LR	LR	HR	IR
G118R	0	2	IR	HR	IR	HR	HR
R263K	5	4	IR	IR	IR	IR	LR
N155H	1	0	LR	LR	LR	HR	HR
P145S	1	0	S	S	S	HR	S
T66I	0	1	S	LR	S	HR	LR
E138K + G140R	0	1	LR	HR	LR	IR	IR
G140R + R263K	0	1	IR	HR	IR	HR	HR

DRMS, drug resistance mutations; BIC, bictegravir; CAB, cabotegravir; DTG, dolutegravir; EVG, elvitegravir; RAL, raltegravir; S, susceptible; LR, low-level resistance; IR, intermediate resistance; HR, high-level resistance. Boxes highlighted in dark red indicate INSTI RAMs conferring high-level resistance to two or more INSTI drugs.

Intermediate-resistance to BIC, CAB, DTG, EVG and RAL were observed in 0.23% (12/5,144), 0.17% (9/5,144), 0.23% (12/5,144), 0.86% (44/5,144), and 0.72% (37/5,144) of the sequences, respectively. Low-resistance was also noted for BIC, CAB, DTG, EVG and RAL, with rates of 0.90, 0.25, 0.90, 0.14 and 0.37%, respectively.

T66I, identified in one individual, is susceptible to BIC and DTG, but it confers low-resistance to CAB and RAL and high-resistance to EVG. E92G, found in 2 individuals, is susceptible to BIC and DTG, however, it confers low-resistance to CAB and RAL and intermediate-resistance to EVG. P145S, detected in one individual, is susceptible to BIC, CAB, DTG and RAL, but it conferes high-level resistance to EVG. (Table 2; Supplementary Figure 2).

### 3.6 Drug resistance to nucleoside reverse transcriptase inhibitors, non-nucleoside reverse transcriptase inhibitors, and protease inhibitors

**Figure 4:**
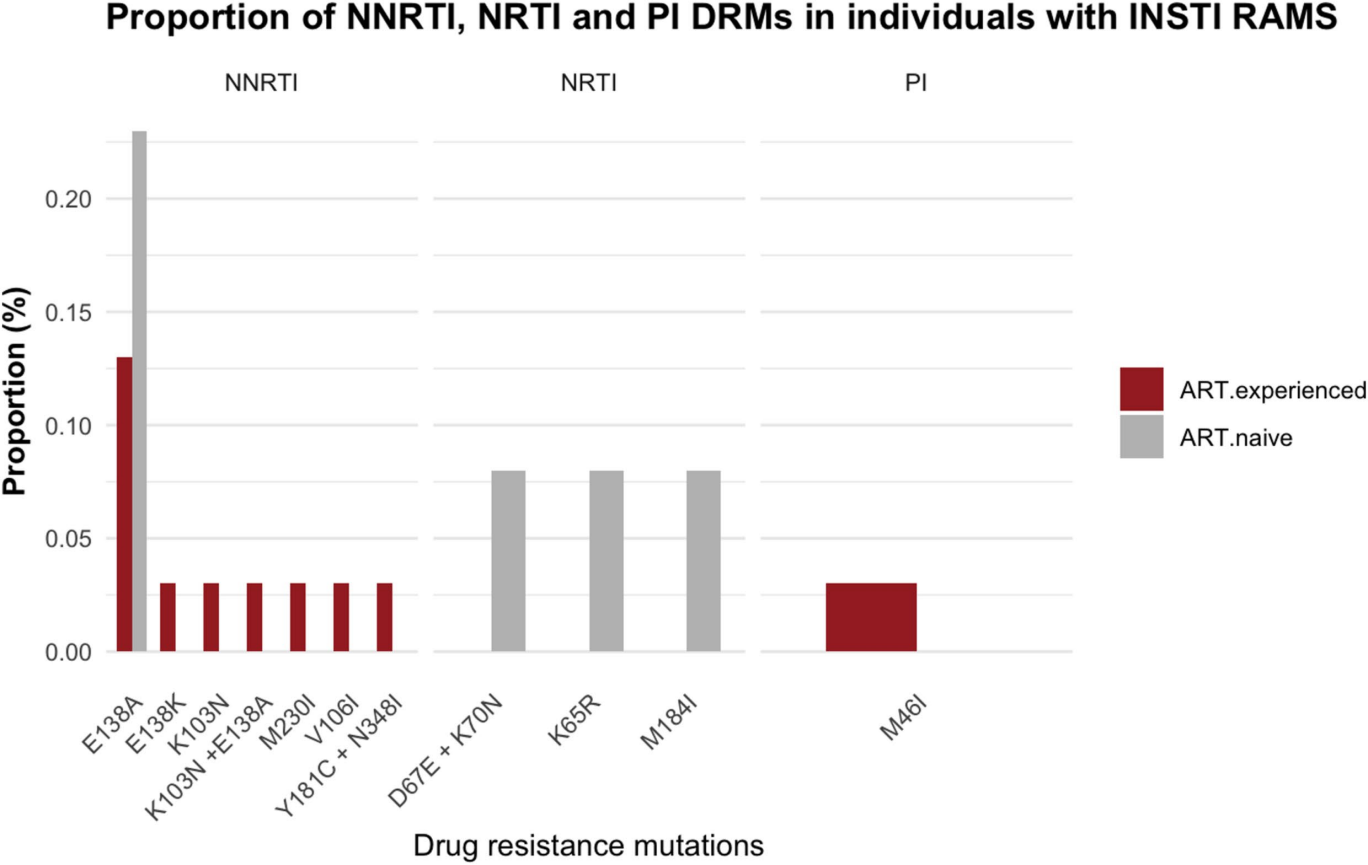
Proportion of drug resistance mutations in individuals with integrase resistance associated mutations among ART-experienced and ART-naïve participants; NRTI, nucleoside reverse transcriptase inhibitors; NNRTI, non-nucleoside reverse transcriptase inhibitors; PI, protease inhibitors.

Within the subset of 57 PLWH with INSTI major resistance mutations, NNRTI-DRMs were the most frequently observed DRMs in 13 individuals (E138A, *n* = 8; E138A + K103N, *n* = 1; M230I, *n* = 1; V106I, *n* = 1; E138K, *n* = 1; K103N, *n* = 1). This was followed by NRTI-DRMs in 3 individuals (D67E + K70N, *n* = 1; K65R, *n* = 1; M184I, *n* = 1), and 1 individual harbored a PI DRM (M46I). (Figure 4; Supplementary Tables 2, 3).

## 4 Discussion

We performed the largest study to date to explore the baseline prevalence of INSTI-RAMs in Botswana near the time of the nationwide rollout of DTG-based ART. We found a favorable low baseline prevalence of INSTI-RAMs of 1.1%, which was slightly higher among ART-naïve individuals (1.64%) compared to ART-experienced individuals (0.93%), suggesting that transmission of INSTI resistant variants was occurring prior to the nationwide rollout of DTG (Kemp et al., 2024). It is possible that the identified INSTI-RAMs were naturally occurring in the HIV-1C in Botswana at a relatively low frequency, or they may have been generated by prior ART use. Although the prevalence of archived INSTI-RAMs was low in Botswana before the rollout of DTG-based first-line therapy, the cross-resistance of these INSTI-RAMs could affect the efficacy of future CAB-based regimens, including CAB PrEP and CAB/RPV LAI.

The prevalence of INSTI-RAMs was similar to that found in other studies from the region (Semengue et al., 2021; Kemp et al., 2024) and from other parts of the world (Chang et al., 2016). The prevalence of INSTI resistance was 1.1% (57/5,144) and this was close to the prevalence in Uganda (1.2%, 6/511; McCluskey et al., 2021). However, this prevalence was slightly higher than that reported in Italy (0.2%) by Rossetti (Rossetti et al., 2018), but notably lower than the prevalence in Poland (8.3%) as documented by Parczewski et al. (2012). The variations in the prevalence of INSTI resistance across different regions can be attributed to differences in sequencing methods, the range of subtypes analyzed, and various patient characteristics, including age and treatment history. Additionally, some studies include an analysis of minority variants, which are low-frequency mutations found in a small percentage of viral genomes, while others do not. Furthermore, discrepancies in defining INSTI resistance, such as considering low-level resistance mutations or restricting the analysis to intermediate and high-level INSTI resistance, contribute to the observed variations in prevalence across different regions.

In the current study, a total of 59 major INSTI-RAMs were identified in 57 individuals. These mutations included E138A (*n* = 5), E92G (*n* = 2), E92Q (*n* = 2), G118R 9 (*n* = 2), G140R (*n* = 32),G140R + R263K (*n* = 1), N155H (*n* = 1),P145S (*n* = 1), R263K (*n* = 9),T66I (*n* = 1),138 K + G140R (*n* = 1), as well as eight accessory INSTI-resistant mutations A128T (*n* = 1) and G163R (*n* = 7). These mutations confer varying levels of resistance to the following INSTIs: BIC, DTG, CAB, EVG and RAL. Interestingly, there is a difference in the prevalence of INSTI resistance among ART naïve participants when compared to those who are on ART, with rates of 1.64 and 0.93% respectively, (*p* = 0.03). These findings emphasize the significance of early detection and management of drug resistance to ensure the effectiveness of ART regimens and to prevent the development and spread of resistance. This suggests that resistance mutations may be more prevalent before treatment initiation and can diminish with effective suppression. The finding that no resistance was detected in participants with virological failure is particularly surprising and warrants further investigation.

We observed an increased occurrence of G140R of 0.62% in ART-naïve and 0.65% in ART-experienced compared to previous reports (Diaz et al., 2023; Branda et al., 2024; Semengue et al., 2021). Even though the percentage increase seems small in a large population, this could translate into a significant number of participants. G140R is an uncommon mutation associated with diminished replication capacity. It has been documented in only one individual receiving CAB treatment (Orkin et al., 2020). Although G140R is considered a rare mutation, a single case was reported in a person living with HIV who experienced treatment failure with CAB (Orkin et al., 2020), resulting in a 6.7-fold reduction in CAB susceptibility (Jeffrey et al., 2022). Consequently, there is a possibility that this mutation could be selected by DTG in cases of virologic failure. Considering the structural and functional resemblances between DTG and CAB, the G140R may also reduce the effectiveness of DTG. As a result, this means that individuals with this mutation might have limited treatment options if they experience virologic failure on DTG. Further research is needed to fully understand the impact of the G140R mutation on DTG and other INSTIs.

The observed resistance levels indicate a concerning degree of resistance to CAB, with 36 individuals exhibiting high-level resistance (28 ART experienced and 8 ART naïve). High-level resistance to CAB may reduce the effectiveness of DTG, CAB PrEP, and LAI CAB/RPV (Parikh et al., 2022). CAB has a lower genetic barrier to resistance than BIC or DTG, despite having a higher barrier to resistance than EVG or RAL. The lower genetic barrier to resistance of CAB can make it easier for specific resistance mutations to emerge and accumulate, leading to high-level resistance to CAB (Oliveira et al., 2018). The detection of high-level drug resistance to CAB in both ART naïve and ART experienced individuals highlights the need for continued surveillance to address the potential impact of drug resistance in the treatment of HIV-1 positive individuals. Similarly, eight individuals, evenly distributed between ART-naïve and ART-experienced participants, displayed high-level resistance to EVG (4 ART naïve and 4 ART experienced). Three participants demonstrated high-level resistance to RAL with one being ART naïve individual and the other two were ART-experienced individuals who had not been treated with RAL. It is noteworthy that no high-level resistance was detected for DTG or BIC. DTG and BIC are the most recent INSTIs and have been associated with lower incidence of high-level resistance mutations due to their higher genetic barrier to resistance compared to earlier generation INSTIs such as EVG and RAL (Max, 2019; Oliveira et al., 2018). The absence of high-level resistance to DTG and BIC underscores their potential utility in managing HIV infections.

Among the subset of 57 sequences showing major INSTI RAMs, NRTI DRMs were observed in 4 participants (3 ART-naïve and 1 on ART with viral suppression), NNRTI DRMs in 13 participants (3 ART-naïve and 10 on ART with viral suppression), and 1 participant on ART with viral suppression had a PI DRM. The accumulation of DRMs to PI, NRTI and NNRTI has the potential to reduce the effectiveness of antiretrovirals when used in combination with INSTI. This increases the risk of a functional INSTI monotherapy and contributes to the onset of INSTI resistance (Naeger et al., 2016; Underwood et al., 2022). Our data show that NNRTI resistance mutations are the most common DRMs amongst participants presenting with INSTI DRMs in the absence of INSTI exposure. These results highlight the need to closely monitor INSTI-RAMs in individuals who experience virologic failure on NNRTI based ART with NNRTI resistance mutations.

The proviral DNA sequencing possesses the capability to detect archived DRMs that are otherwise not detectable in plasma samples due to low viral load (Singh et al., 2016; Sotillo et al., 2018). Therefore, these data should be interpreted with caution as the clinical implications of HIV DRMs detected from proviral DNA compartment are not well understood (Ho et al., 2013). Studies investigating the clinical impact of these HIV DRMs are warranted.

## 5 Limitations

Strengths of our study included use of next generation sequencing for comprehensive analysis of DRMs across the entire pol gene, including those associated with INSTIs, NNRTI, NRTI and PI. The study’s potential limitations encompassed the reliance on self-reported ART naïve status among certain participants. Additionally, we did not have information on the previous ART regimens used by participants in the BCPP cohort. This limitation raises the possibility that some individuals classified as ART-naïve might be ART-experienced, potentially leading to misclassification of drug resistance. Finally, a notable limitation of this data is the lack of analysis of HIV minority variants. The majority rule consensus was applied to assemble sequences generated by next generation sequencing (NGS; Novitsky et al., 2020). The lack of analysis for HIV minority variants could be a limitation of our study as we could have underreported the prevalence of the INSTI-RAMs. Overlooking their presence could impact the overall findings. The amplification and sequencing of proviral DNA can also overpredict the prevalence of DRMs owing to hypermutations (Li et al., 2021). Proviral DNA sequencing has been associated with overestimating archived DRMs due to the presence of hypermutations, which can result in false positives for drug resistance (Li et al., 2021). Further studies comparing DRMs in plasma and proviral DNA from antiretroviral-naïve individuals are needed to address these discrepancies.

## 6 Conclusion

In conclusion, our findings indicate that resistance to INSTIs was low among both ART naïve and ART experienced individuals in Botswana near the time of the nationwide DTG rollout. The prevalence rates of major INSTI resistance mutations in ART-naïve and ART-experienced were 1.64 and 0.93%, respectively. While these archived proviral resistance rates were low, they are expected to increase in the current era of nearly universal DTG use, underscoring the need for vigilant monitoring to detect and address the potential emergence of DTG resistant strains over time.

## Data Availability

Publicly available datasets were analyzed in this study. This data can be found here: HIV-1 sequences are available on request through the PANGEA consortium (www.pangea-hiv.org). BCPP data are available at https://data.cdc.gov/Global-Health/Botswana-Combination-Prevention-Project-BCPP-Publi/qcw5-4m9q.

## Footnotes

^1^http://www.hiv.Ianl.gov

## Group members for PANGEA Consortium

Phylogenetics and Networks for Generalized Epidemics in Africa, London, United Kingdom.
