# Supplementary material for: Low prevalence of archived integrase strand transfer inhibitors resistance associated mutations in Botswana before the roll out of dolutegravir based first line antiretroviral therapy
Source: Front Microbiol. 2024 Oct 24;15:1482348. doi: 10.3389/fmicb.2024.1482348 (PMC11540625; doi:10.3389/fmicb.2024.1482348)
Supplement: Supplementary file 1 [file Table_1.DOCX]

**Supplementary Table 1:** Intervention and Control Communities in the Botswana Combination Prevention Project

| **Interventions communities** | **Control communities** |
| --- | --- |
| Digawana | Ranaka |
| Otse | Molapowabojang |
| Lentsweletau | Letlhakeng |
| Oodi | Bokaa |
| Mmankgodi | Mmathethe |
| Lerala | Sefhophe |
| Maunatlala | Ramokgonami |
| Shoshong | Mmadinare |
| Tati Siding | Metsimotlhabe |
| Nkange | Sebina |
| Mathangwane | Mmandunyane |
| Gweta | Rakops |
| Gumare | Shakawe |
| Sefhare | Tsetsebjwe |
| Masunga | Nata |
