# Supplementary material for: Low prevalence of archived integrase strand transfer inhibitors resistance associated mutations in Botswana before the roll out of dolutegravir based first line antiretroviral therapy
Source: Front Microbiol. 2024 Oct 24;15:1482348. doi: 10.3389/fmicb.2024.1482348 (PMC11540625; doi:10.3389/fmicb.2024.1482348)
Supplement: Supplementary file 2 [file Table_2.docx]

**Supplementary Table 2** NRTI, NNRTI and PI resistance mutations among PLWH with INSTI resistance mutations

| **DRMs** | | **ART status** | | **NNRTIs resistance level** | | | | |
| --- | --- | --- | --- | --- | --- | --- | --- | --- |
| **NNRTI DRMs** |  | **ART-naïve**  **n =1281, (%)** | **ART-experienced**  **n = 3863 (%)** | **DOR** | **EFV** | **ETV** | **NVP** | **RPV** |
|  | E138A | 3 (0.23) | 5 (0.13) | S | S | PLLR | S | LLR |
|  | E138K | - | 1 (0.03) | S | PLLR | PLLR | PLLR | IR |
|  | K103N | - | 1 (0.03) | S | HLR | S | HLR | S |
|  | M230I | - | 1 (0.03) | LLR | LLR | LLR | IR | IR |
|  | V106I | - | 1 (0.03) | PLLR | S | PLLR | PLLR | PLLR |
|  | Y181C + N348I | - | 1 (0.03) | PLLR | IR | IR | HLR | PLLR |
|  | K103N +E138A | - | 1 (0.03) | S | HLR | PLLR | HLR | LLR |
| **NRTI DRMs** |  |  |  | **NRTIs resistance level** | | | | |
|  |  |  |  | **AZT** | **FTC** | **3TC** | **TDF** | |
|  | D67E + K70N | 1 (0.08) | - | LLR | PLLR | PLLR | LLR | |
|  | K65R | 1 (0.08) | - | LLR | HLR | HLR | HLR | |
|  | M184I | 1 (0.08) | - | S | HLR | HLR | S | |
| **PI DRMS** |  |  |  | **PI resistance level** | | | | |
|  |  |  |  | **AZV** | **DRV/r** | **LPV** | | |
|  | M46I | - | 1 (0.03) | PLLR | S | PLLR | | |

DRMs, drug resistance mutations; NNRTI, nucleoside reverse transcriptase inhibitors; NRTI, nucleoside reverse transcriptase inhibitors, PI, protease inhibitors, DOR, doravirine; EFV, efavirenz; ETV, etravirine; NVP, nevirapine; RPV, rilpivirine; S, susceptible; LLR, low-level resistance; IR, intermediate resistance; PLLR, Potential low-level resistance; HLR, High-level resistance.
