# Supplementary material for: Low prevalence of archived integrase strand transfer inhibitors resistance associated mutations in Botswana before the roll out of dolutegravir based first line antiretroviral therapy
Source: Front Microbiol. 2024 Oct 24;15:1482348. doi: 10.3389/fmicb.2024.1482348 (PMC11540625; doi:10.3389/fmicb.2024.1482348)
Supplement: Supplementary file 3 [file Table_3.docx]

**Supplementary Table 3** Fifty-seven individuals with INSTI drug resistance mutations and other mutations

| **seq_id** | **art_status** | **INSTIMajor** | **NRTIs** | **NNRTIs** | **Pis** |
| --- | --- | --- | --- | --- | --- |
| BW_2013.87_066_12340015_1_PANGEA_JUN2016 | ART naive | G140R | None | None | None |
| BW_2016.34_066_13430008_3_fc04072 | ART naive | E138K | None | None | None |
| BW_2014.36_066_14100036_5_fc03611 | ART naive | R263K | None | None | None |
| BW_2014.76_066_15120039_3_PANGEA_JUN2016 | On ART | G140R | None | M230I | None |
| BW_2014.69_066_16260010_3_PANGEA_JUN2016 | ART naive | G140R | None | E138A | None |
| BW_2016.55_066_16920470_6_fc03611 | ART naive | R263K | D67E, K70N | E138A | None |
| BW_2014.87_066_17250019_2_PANGEA_JUN2016 | ART naive | G140R | None | None | None |
| BW_2017.3_066_191400023_6_fc04500 | On ART | T66I | None | None | None |
| BW_2016.11_066_19270002_2_fc04072 | ART naive | E92Q | None | None | None |
| BW_2015.08_066_20100026_1_fc03322 | On ART | G140R | None | None | None |
| BW_2015.09_066_20160014_6_PANGEA_JUL2016 | ART naive | N155H | K65R | None | None |
| BW_2015.06_066_20190017_0_PANGEA_JUL2016 | ART naive | E92Q | None | None | None |
| BW_2015.09_066_20320026_5_fc02882 | On ART | G140R | None | None | None |
| BW_2017.25_066_204500009_5_fc04938 | On ART | G140R | None | V106I | None |
| BW_2017.51_066_204800115_7_fc04938 | ART naive | G140R | None | None | None |
| BW_2015.06_066_20850007_2_fc02882 | On ART | G140R | None | None | None |
| BW_2016.51_066_21930458_3_fc03746 | On ART | G118R | F227L | None | None |
| BW_2016.61_066_21930512_1_fc04229 | On ART | G140R | None | E138K | None |
| BW_2016.69_066_21930573_6_fc03611 | ART naive | G140R | None | None | None |
| BW_2016.86_066_21930670_5_fc03746 | ART naive | R263K | None | None | None |
| BW_2016.91_066_21930703_3_fc03633 | ART naive | G140R | None | None | None |
| BW_2015.13_066_22400025_3_PANGEA_JUL2016 | ART naive | E92G | None | None | None |
| BW_2015.19_066_23540008_1_f950 | On ART | G140R | None | E138A | None |
| BW_2017.52_066_236300016_4_fc04938 | On ART | G140R | None | None | None |
| BW_2017.59_066_236400061_9_fc04938 | On ART | G140R | None | None | None |
| BW_2016.49_066_23910441_1_fc03322 | On ART | G140R | None | E138A | None |
| BW_2015.2_066_24120016_3_fc03322 | ART naive | R263K | None | None | None |
| BW_2015.19_066_24430012_4_PANGEA_JUL2016 | ART naive | E92G | None | None | None |
| BW_2015.25_066_25100008_2_fc03209 | On ART | G140R | None | None | None |
| BW_2015.3_066_25480055_5_f950 | On ART | G140R | None | K103N, E138A | None |
| BW_2015.23_066_26270003_3_fc03037 | On ART | R263K | None | None | None |
| BW_2016.47_066_28350004_3_fc04072 | ART naive | R263K | None | None | None |
| BW_2015.92_066_30930229_0_fc04041 | On ART | E138K | None | None | None |
| BW_2016.13_066_30930321_1_fc04041 | On ART | G140R,R263K | None | None | None |
| BW_2016.3_066_30930357_2_fc04159 | On ART | G118R | None | None | None |
| BW_2015.46_066_31280002_4_fc02746 | On ART | G140R | None | None | None |
| BW_2016.72_066_31920597_1_fc03928 | ART naive | G140R | None | None | None |
| BW_2015.52_066_33310015_3_bpf_DEC2016 | On ART | R263K | None | None | None |
| BW_2015.56_066_34100030_5_2017JAN | ART naive | E138K | None | None | None |
| BW_2017.87_066_351500600_0_fc04229 | On ART | G140R | None | None | None |
| BW_2015.61_066_35390003_4_2017JAN | ART naive | G140R | M184I | None | None |
| BW_2015.67_066_35400048_4_2017JAN | On ART | G140R | None | None | None |
| BW_2015.65_066_36110026_0_fc02746 | On ART | G140R | None | None | None |
| BW_2017.87_066_364200036_3_fc04622 | On ART | R263K | None | None | None |
| BW_2015.65_066_36550011_0_fc02746 | On ART | R263K | None | None | None |
| BW_2017.85_066_365800001_8_fc04860 | On ART | G140R | None | None | None |
| BW_2017.94_066_366300072_2_fc04072 | On ART | G140R | None | None | None |
| BW_2018.11_066_36890001_0_fc05127 | On ART | G140R | None | None | M46I |
| BW_2015.81_066_37370024_5_fc03152 | On ART | G140R | None | K103N | None |
| BW_2015.81_066_38100028_0_fc03152 | On ART | G140R | None | None | None |
| BW_2015.73_066_39430027_6_f950 | ART naive | P145S | None | E138A | None |
| BW_2015.74_066_40150042_1_fc03746 | On ART | E138K,G140R | None | E138A | None |
| BW_2015.71_066_40270009_2_fc03746 | On ART | E138K | None | None | None |
| BW_2015.73_066_40530027_5_fc03746 | On ART | G140R | None | None | None |
| BW_2015.73_066_40630028_4_fc03928 | On ART | E138K | None | E138A | None |
| BW_2015.76_066_39190046_6_f950 | On ART | G140R | None | None | None |
| BW_2015.85_066_38380037_2_fc03268 | On ART | G140R | None | E138A | None |
