# Supplementary figures and images for: Low prevalence of archived integrase strand transfer inhibitors resistance associated mutations in Botswana before the roll out of dolutegravir based first line antiretroviral therapy
Source: Front Microbiol. 2024 Oct 24;15:1482348. doi: 10.3389/fmicb.2024.1482348 (PMC11540625; doi:10.3389/fmicb.2024.1482348)

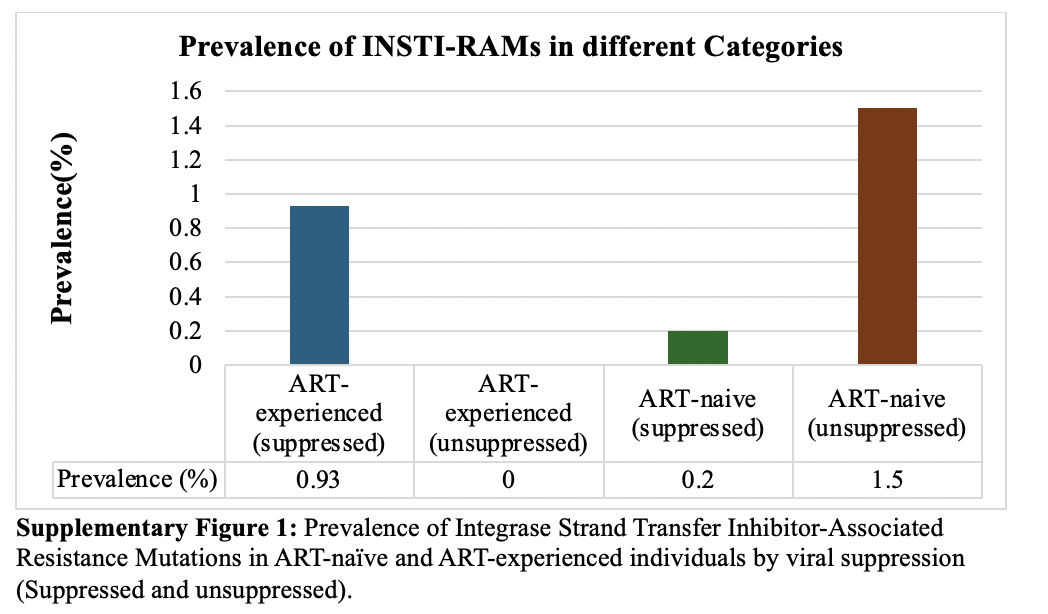

Supplement: Supplementary file 4 [file Image_1.tiff]

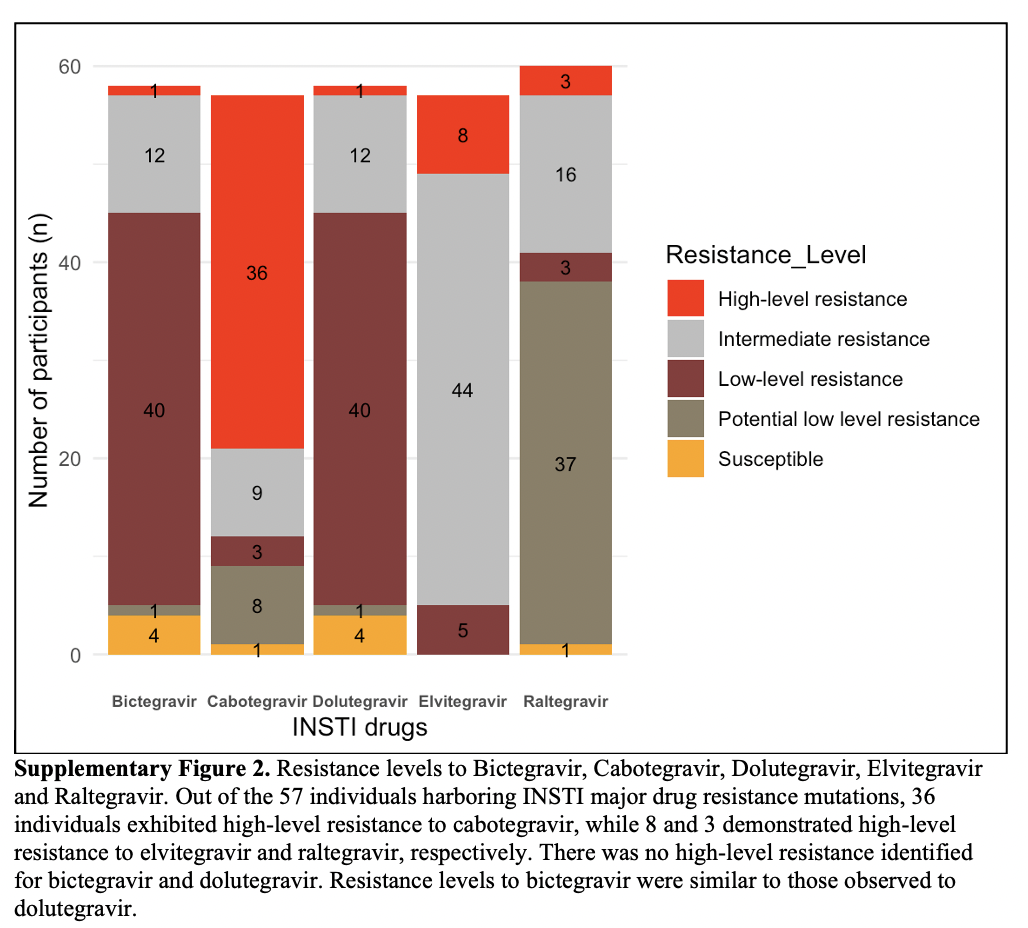

Supplement: Supplementary file 5 [file Image_2.tiff]
